# Multifaceted roles of *Candida albicans* and *Streptococcus mutans* in contributing to polybiofilm infections in early childhood caries

**DOI:** 10.3389/fcimb.2025.1625103

**Published:** 2025-06-27

**Authors:** Anto Benignus Francis, Rajendra Prasad Settem, Moghanram Jeyamoorthy, Venkata Harshith Nuthangi, Ashu Sharma, Satish Kumar Rajasekharan

**Affiliations:** ^1^ Department of Biotechnology, School of Bioengineering, SRM Institute of Science and Technology, Kattankulathur, Chengalpattu District, Tamil Nadu, India; ^2^ University at Buffalo, State University of New York, Buffalo, NY, United States

**Keywords:** biofilm, ECC, polybiofilms, Candidia albicans, Streptococcus mutans

## Abstract

This succinct article addresses the multifaceted interactions between the fungal organism *Candida albicans* and the Gram-positive bacterium *Streptococcus mutans* in the development of oral biofilms and pathobiology of oral diseases. *S. mutans* is considered to be a major pathogen in the development of dental caries. It is often found to interact with *C. albicans* in oral infection settings. The interaction of these organisms is often mediated via the binding of Glucosyltransferase (GtfB) enzyme secreted by *S. mutans* to *C. albicans* surface proteins Als1 and Hwp1. During these interactions, both *C. albicans* and *S. mutans* exhibit increased gene regulatory activity, leading to the modulation of virulence attributes and adaptation to environmental changes. This results in the strong attachment of the species to tooth surfaces and increased resistance of the mixed species biofilms to external factors. Mechanistically, intercellular communication between these species in mixed biofilms through quorum sensing and production of exoenzymes such as glucosyltransferases account for the synergy and modulation of their virulence attributes. Specifically, these mixed-species biofilms exhibit increased acid production and enhanced resistance to antimicrobial agents. Understanding these complex interkingdom pattern of interactions is essential to develop efficient therapeutic approaches against biofilm-associated oral infections. The review also highlights probiotic strategies to interfere with these interkingdom interactions to combat oral diseases like early childhood caries (ECC).

## Introduction

**Figure 1:**
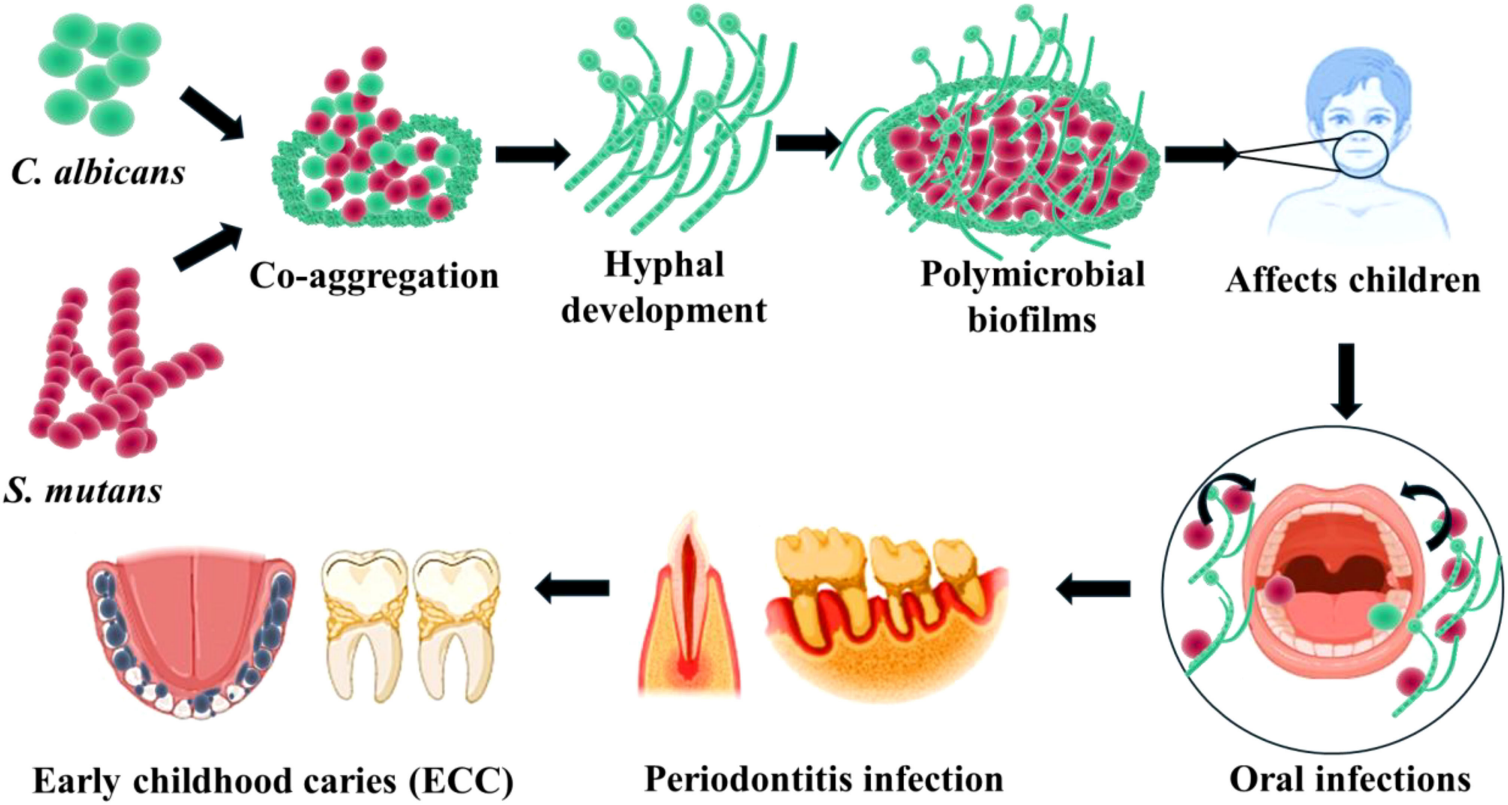
Cross-kingdom interactions of *S. mutans* and *C. albicans* in oral microenvironment impact the development of Early childhood caries and various oral infections.

The majority infectious diseases worldwide develop from the formation of biofilms on mucosal surfaces by specific organisms. Early Childhood Caries (ECC) being one of the most common biofilms associated tooth infection, notably increasing among preschool children. In ECC, *S. mutans* and *C. albicans* are oral pathogens which form mixed species polymicrobial biofilms over the enamel and dental surfaces (Shirtliff et al., 2009). *S. mutans* has been recognized as one of the major etiologic agents of caries. It can rapidly form plaque biofilms on tooth surfaces when exposed to sucrose and induce pathogenicity. *S. mutans-*secreted glucosyltransferases (Gtfs) can utilize sucrose to produce an insoluble exopolysaccharide (EPS) matrix, which constitutes the primary building block of biofilms over the enamel (Bowen and Koo, 2011) (**Figure 1**). The glucan polymer formation increases cohesion between S. mutans and Candida and additionally present binding sites for other microorganisms to adhere and colonize (Falsetta et al., 2014). Specifically, in the presence of sucrose *S. mutans* adheres strongly to fungal cells. Such interactions lead to virulent forms of organisms due to the formation of hypervirulent biofilms on oral surfaces, leading to decaying of tooth surfaces (Peleg et al., 2010). Moreover, *C. albicans* by adhering to organisms increases its carriage and infectivity.

### Role of oral pathogens in Early childhood caries

Early childhood caries (ECC) is one of the common diseases affecting children worldwide (Lu et al., 2023). While acid-producing *S. mutans* is the primary etiological agent in initiating caries, *C. albicans* is often observed coexisting in the dental plaque of children with ECC, particularly in cases of Severe ECC. In these dental plaques, *Candida* and *Streptococcus* spp. form mixed biofilms on the surface of teeth, forming a thin layer of organic membrane. In addition, the surface proteins of *S. mutans* can also efficiently bind to other microbes, enhancing their colonization and initiating proliferation and the formation of complex multispecies polymicrobial biofilms. The process of multi-species biofilm formation is thought to be initiated by *S. mutans* via two different pathways: sucrose dependent and sucrose independent. In this, the sucrose-dependent pathway includes the production of glucosyl transferases GtfB, GtfC, and GtfD. In the presence of sucrose, the glucosyltransferases produce water-soluble or water-insoluble glucans the Gtfs possess sucrose-dependent activity that causes glycosidic bond breakage and releases fructose and glucose. The glucose is then linked to a developing glucan polymer (Lemos et al., 2019).

**Figure 2:**
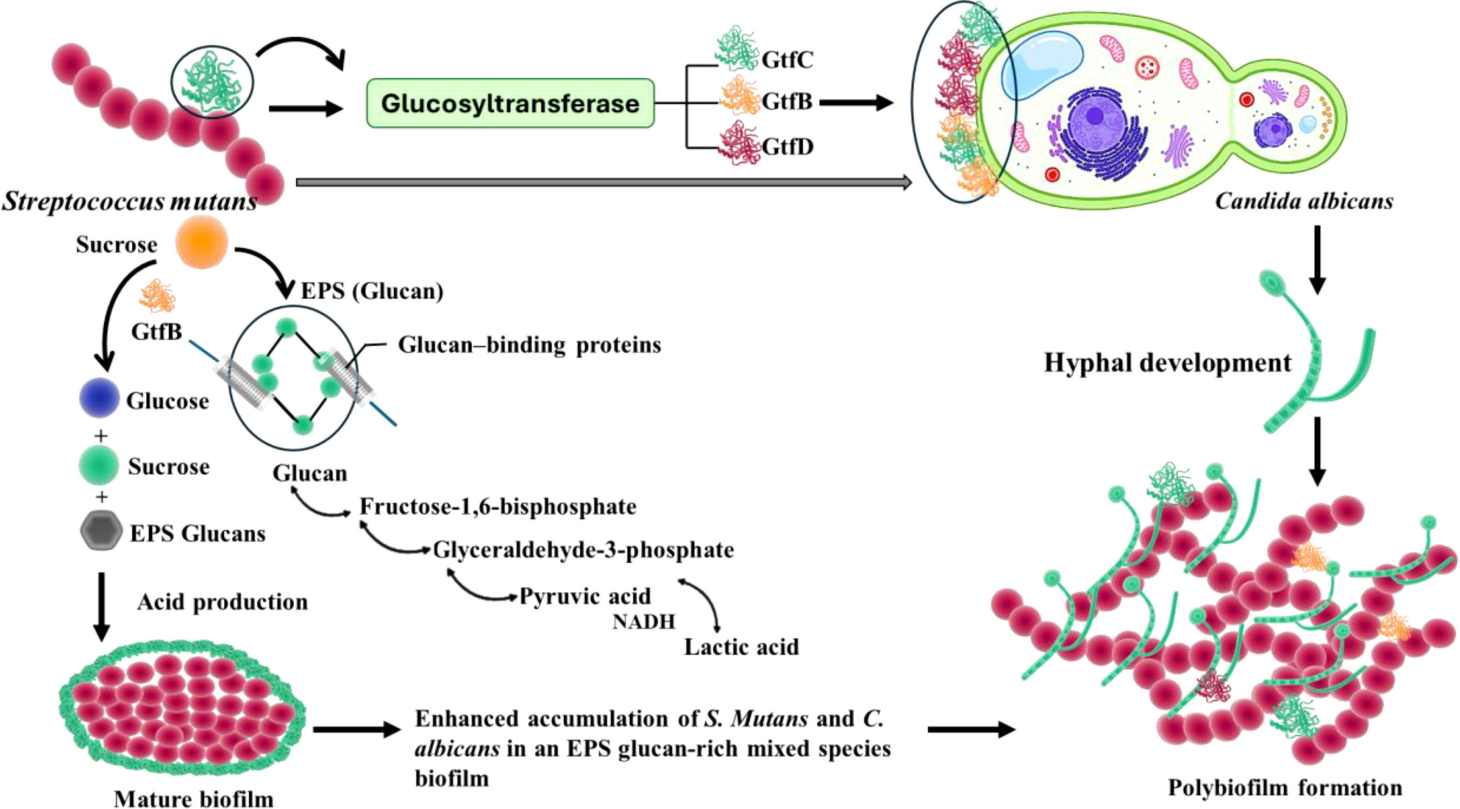
The interaction of *S. mutans* with *C. albicans* influences the virulence of *C. albicans*, by the secreted molecules and cell surface molecules by *S. mutans*, which also secretes Gtfs that will be attached to *C. albicans* and plays a crucial role in pathogenesis.

Here, GtfB is the major producer of α-1, 3 linked insoluble polysaccharide glucan, the major component of biofilm matrix, which provides adhesion sites and accumulation sites for other microbes to attach and initiate the formation of polymicrobial biofilms (**Figure 2**) (Cui et al., 2021, Hwang et al., 2017). The glucosyltransferase GtfB, produced by *S. mutans*, produces glucans from sucrose. The role of GtfB is to promote the adhesion of the EPS matrix over the enamel, causing the formation of biofilm. Gtfs will also be adsorbed to the surface of other microorganisms and converted into glucan producers (Bowen and Koo, 2011). The production of glucans on the tooth surface further promotes biofilm formation by increasing the adherence of *S. mutan*s mediated by glucan binding proteins expressed by *S. mutans* (Matsumoto-Nakano, 2018). GtfB also promotes the aggregation of bacterial species and enables its growth in the hostile oral environment. Moreover, GtfB fosters coaggregation with other pathogenic species, including *C. albicans*, thus causing the formation of dual-species biofilm (Kulshrestha and Gupta, 2022). *S. mutans* also possesses multiple high affinity adhesins that enable the organism to adhere to tooth surfaces in the absence of sucrose. In this regard, the dual antigen I/II (also known as P1, SpaP, or Pac) is a multifunctional adhesin that can mediate bacterial attachment to the salivary pellicle formed on tooth surfaces and other bacteria (Brady et al., 2010).

*S. mutans* acts as one of the leading causing factors of ECC by producing organic acids via carbohydrate metabolism. Oligosaccharides from the breakdown of carbohydrate polymers are primarily transported into the cells by ATP-binding cassette (ABC) transporters, whereas monosaccharides (glucose and fructose) and disaccharides (sucrose) are predominantly taken up by the phosphoenolpyruvate dependent phosphotransferase transporters (PTS). In the cytoplasm, phosphorylated sugars are then processed to fructose-6-phosphate (Fru-6-P) and fermented into organic acids, mainly lactic acid, via glycolysis. The accumulation of these acids in the oral microenvironment reduce the pH, which leads to the major cause of demineralisation of the tooth surface. Acid resistance therefore is an immense attribute and survival tool of *S. mutans* (Lemos et al., 2019). It has also been shown that *C. albicans* can also produces acids by metabolizing carbohydrates, further exacerbating low pH of the oral environment.

### Bacteria-yeast interplay

Several adhesion proteins (adhesins) mediate the attachment of microbial species to the surfaces of the oral cavity, especially to the tooth surface for the development of pathogenic biofilms of *C. albicans* and *S. mutans*. *C. albicans* possesses key adhesins, such as Als1, Als3, and Hwp1 that are involved in the attachment to host tissues and formation of hyphal structures that allow the invasion and development of biofilm. *C. albicans* interacts with bacterial species such as *S. mutans* through these adhesins to establish mixed-species biofilms and ensure the persistence of infection. In the process, *S. mutans* promotes biofilm thickening and complexity (Lu et al., 2023). Here, *S. mutans* adhesins, such as SpaP and Epa1, are involved in adhesion of the bacterium to the tooth surface and assembly of the biofilm. Ultimately, the biofilm community surrounded by a protective EPS meshwork induces dental caries by promoting acid production.

Interestingly, the expression of adhesin-expressing genes in *S. mutans* is upregulated by *C. albicans*, suggesting that the presence of fungal cells might enhance the capacity of *S. mutans* cells to colonize and form biofilms. Further, the interaction between these species occurs through a process called quorum sensing, which is a chemical communication system that induces the gene expression of adhesive molecules in *C. albicans* and *S. mutans* (Sztajer et al., 2014). Still, the protein family of *Als* continues to be central to the pathogenicity of *C. albicans*, which causes tissue invasion, immune evasion, and biofilm formation. Those interactions between adhesion proteins of *S. mutans*, such as *SpaP* and *Epa1* enhance the complexity of oral biofilms and make it tough to disrupt the targeted synergistic interactions and prevention of biofilm diseases (Kriswandini and Almas, 2023). During such interaction, these *S. mutans* and *C. albicans* will evolve as a complex network of regulatory mechanisms to boost cariogenic virulence and will modulate tolerance upon stress changes in the external environment (Li et al., 2023).

### Polymicrobial biofilm infections

*S. mutans* and *C. albicans* form dual-species biofilms that share a common matrix, influencing pathogenicity and virulence. For structural integrity and resilience of biofilms, extracellular polymeric substances provide support and stability. It has been shown that production the rate of synthesis of the extrapolysaccharide glucan substance (EPS) is significantly higher in mixed dual-species biofilms than in mono-species biofilms (Falsetta et al., 2014). Such increased production of EPS promotes the retention of nutrients and assistance in metabolic cross-feeding, causing an increased pathogenic capacity of both organisms. The extracellular polysaccharides matrix also allows each resident to collaborate and interact with its neighbouring species so that it can survive in such a hostile environment (Weiland-Bräuer, 2021)Nutrients are the major determining component in the colonization of microbial species. Metabolic communication involving excretion by one organism and utilization by another is a common feature in mixed species oral biofilms. For instance, early colonizer species can secrete short-chain acids such as lactate, pyruvate, and acetate through sugar metabolism, which can form a high energy source for the late colonizers. In addition to the contact-dependent microbial communication (signalling), intercellular communication (signalling) can occur via secreted diffusible molecules (Guo et al., 2024) (**Table 1**). *C. albicans* can induce several genes in *S. mutans*, such as gtfB responsible for glucan synthesis, a major component of insoluble EPS. This improves antimicrobial resistance and protects *C. albicans* from antifungal agents like fluconazole (Li et al., 2023). This environment improves metabolic interactions between species, where *S. mutans* could influence the growth of *C. albicans* in a similar way pathogenicity and virulence of these biofilms increased making it difficult to treat (Li et al., 2023).

**Table 1:** Infections caused in diverse population by the pathogenic bacteria and yeast in oral microbiome due to their interactions.

Population	Association	Infection	Reference
Child (0–5 y)	Meta-analysis: *C. albicans* is significantly linked to ECC.	ECC	(Man et al., 2025)
Adult (18–60 y)	Cordycepin interferes with *S. mutans* sugar metabolism and biofilm formation, suggesting its use to modulate cariogenic synergy with *C. albicans*.	Dental caries	(Shao et al., 2025)
Children (< 4 y)	Interaction leads to the demineralisation of tooth issues.	ECC	(Jin et al., 2024)
Adult (>50 y)	Co-colonization on dentures leads to inflammation and fungal infection.	Denture stomatitis	(Perić et al., 2024)
Child (0–6 y)	Systematic meta-analysis reveals a strong positive association between co-presence of *C. albicans* and *S. mutans* in children with ECC.	ECC.	(Khachatryan et al., 2024)
Adult (5–15 y)	Metabolic cooperation between *C. albicans* and *S. mutans* boosts acid production, reinforcing their persistence in dental plaque.	Dental caries	(Guo et al., 2023)
Children (3–5 y)	Interaction leads to low growth parameters and sleep disorders.	ECC	(Lu et al., 2023)
Infants (0–1 y)	Early *C. albicans* predicts *S. mutans* colonization and higher caries risk.	ECC	(Menon et al., 2022)
Adult (18–40 y)	Asymptomatic *C. albicans* carriage linked to increased caries experience.	Dental caries	(Eidt et al., 2020)
Children (3–5 y)	Interaction of *C. albicans-S. mutans* in children with ECC.	ECC	(Bachtiar and Bachtiar, 2018)
Children (6–12 y)	Acidogenic co-biofilm formation enhances enamel demineralization.	Dental caries	(Xiao et al., 2018)
Children (2-11y)	Had dental caries due to the interaction.	Dental caries	(Metwalli et al., 2013)
Children (5y)	High plaque levels of *C. albicans* and *S. mutans* correlate with carious lesions.	ECC	(Sridhar et al., 2020)
Adult (20–60 y)	Synergistic mucosal biofilms of *C. albicans* and *S. mutans* worsen inflammation.	Oral Candidia-sis	(Diaz et al., 2012)

### Role of *S. mutans* Glucosyl transferases Gtfs in triggering *C. albicans* virulence

Glucosyl transferases (GtfB, GtfC, and GtfD) expressed by *S. mutans* play critical roles in *C. albicans-S. mutans* mixed species biofilm formation and caries pathogenesis (**Figure 2**). As indicated above, the biofilm formation and plaque development due to *S. mutans* and *C. albicans* are influenced by the exoenzymes such as GtfB, and the adhesins such as Als1 and Als3. The role of Gtf enzymes is to assist in enhanced biofilm formation by enabling *S. mutans* to colonize by accumulating EPS. Gtfs share some similarities in structure, but they possess various unique functions. Among these Gtfs, GtfB produces glucans, which facilitate the attachment of microbes and alter the biofilm structure by enhancing the interaction with various oral microbes (Ellepola et al., 2019). In such dual biofilm formation, *S. mutans* is involved in the metabolism of carbohydrates, degradation of pyruvate, also the production of acetate and ethanol and influences the electron transport chain and the tricarboxylic acid cycle. During the interaction of *S. mutans* and *C. albicans*, *C. albicans* utilizes glucose predominantly but it can’t metabolize sucrose efficiently. But *S. mutans* is able to convert the remaining sucrose into glucans through the Gtf system. This influences the growth of *C. albicans* and improves the production of excess acids in the oral cavity. Further, the exoenzyme GtfB from S. mutans can bind to the mannan layer of the cell wall of *C. albicans*. Such interactions affect the colonization of *C. albicans* in the oral cavity.

## Conclusion

From this review, we conclude that the oral opportunistic pathogen *C. albicans* and *S. mutans*, due to their interaction, play a major role in the pathogenesis and formation of various oral diseases through distinct mechanisms. Also, the interaction between these species proves that it is a major cause for the formation of ECC worldwide. This observation serves a critical role in proving that the extracellular matrix plays a crucial role in the formation of biofilm and leads to the formation of mixed-species poly-biofilms due to the interaction of *S. mutans* and *C. albicans*. In addition, further studies are exploring the anti-*Candida* therapies possible in the treatment of ECC and involving the need to develop inhibition agents that inhibit GTFs production during the interaction with *S. mutans*.
